# TCM Prescription Generation via Knowledge Source Guidance Network Combined with Herbal Candidate Mechanism

**DOI:** 10.1155/2023/3301605

**Published:** 2023-01-03

**Authors:** Jiaxin Hou, Ping Song, Zijuan Zhao, Yan Qiang, Juanjuan Zhao, Qianqian Yang

**Affiliations:** ^1^College of Information and Computer, Taiyuan University of Technology, Taiyuan, China; ^2^College of Information, Jinzhong College of Information, Jinzhong, China

## Abstract

Traditional Chinese medicine (TCM) prescriptions have made great contributions to the treatment of diseases and health preservation. To alleviate the shortage of TCM resources and improve the professionalism of automatically generated prescriptions, this paper deeply explores the connection between symptoms and herbs through deep learning technology, and realizes the automatic generation of TCM prescriptions. Particularly, this paper considers the significance of referring to similar underlying prescriptions as herbal candidates in the TCM prescribing process. Moreover, this paper incorporates the idea of referring to the potential guidance information of corresponding prescriptions when model extracts symptoms representations. To provide a reference for inexperienced young TCM doctors when they prescribe, this paper proposes a dual-branch guidance strategy combined with candidate attention model (DGSCAM) to automatically generate TCM prescriptions based on symptoms text. The format of the data used this paper is the “symptoms-prescription” data pair. The specific method is as follows. First, DGSCAM realizes the extraction of key information of prescription-guided symptoms through a dual-branch network. Then, herbal candidates in the form of prescriptions that can treat symptoms are proposed in view of the compatibility knowledge of TCM prescriptions. To our knowledge, this is the first attempt to use prescriptions as herbal candidates in the prescription generation process. We conduct extensive experiments on a mixed public and clinical dataset, achieving 37.39% precision, 25.04% recall, and 29.99% F1 score, with an average doctor score of 7.7 out of 10. The experimental results show that our proposed model is valid and can generate more specialized TCM prescriptions than the baseline models. The DGSCAM developed by us has broad application scenarios and greatly promotes the research on intelligent TCM prescribing.

## 1. Introduction

As a documented summary of the valuable experience of the ancient Chinese people in their struggle against disease [1, 2], traditional Chinese medicine (TCM) is receiving increasing attention and recognition from researchers and scholars around the world [3]. As a key component of TCM, TCM prescriptions realize the conversion between doctors' professional medical skills and patients' treatment needs [4, 5]. In the research field of various diseases, TCM has an inestimable prospect. All kinds of traditional Chinese medicine not only have excellent performance in relieving symptoms but also play a huge role in curing diseases [6–8]. In the fight against the novel coronavirus disease 2019 (COVID-19), traditional Chinese medicines have a remarkable effect on preventing infection and curing mild infections [9–11]. However, the generation and daily application of TCM prescriptions have always been constrained by TCM resources. The small number of TCM health institutions and training colleges, and the strict standards for TCM doctors to become talents, all of which limit the output speed of TCM talents. And these result in a huge discrepancy between the number of TCM doctors and the number of patients [12, 13]. In addition, some newly recruited young doctors are inexperienced and lack standard prescriptions as a reference when prescribing. This makes it difficult for them to determine whether the prescribed prescription is effective. As a result of these problems, it is difficult for both patients and doctors to have a sense of belonging and identity in the field of TCM, which in turn leads to the slow development of TCM.

Faced with these problems, there is an urgent need for an easy way to provide convenience for patients and inexperienced young doctors. With the rise of artificial intelligence [14], some people gradually link the generation of TCM prescriptions with computer technology. Many researchers have conducted standardized and objective researches on the automatic generation of TCM prescriptions [15, 16]. In terms of generating TCM prescriptions from texts, many researchers have tried to explore the correspondence between symptoms and prescriptions using multigraph convolutional networks. Jin et al. [17] proposed an approach to recommend herbal medicines using the MGCN framework to consider the process of the implicit syndrome induction prescription generation. In this study, not only the correlation between symptoms and prescribed herbs was considered but also the intrinsic connection of “symptoms and symptoms” and “herbs and herbs” was studied separately. Yang et al. [18] built a multilayer information fusion graph convolution model by introducing additional information representing herbal attributes. And they used symptom feature representation and herbal feature representation to facilitate prescription generation. In addition, Zhao et al. [19] similarly proposed an MGCN-based prescription recommendation model. This model assisted computer deduction to reproduce the connotation of TCM diagnosis and treatment by focusing on the patient's state elements and syndrome types of symptoms. In addition to the MGCN approach, there are also researchers who use topic models as research ideas. Wang et al. [20] proposed a subject model of TCM theory combined with herbal compatibility, which can better characterize diagnostic and therapeutic processes. Yao et al. [21] proposed a subject model that introduced the background of herbal medicine as domain knowledge to facilitate prescription generation, which used TCM theory to generate prescriptions. Although the above studies have demonstrated the effectiveness of a number of existing computer techniques in TCM prescription generation, the MGCN approaches in these studies did not take into account the correlation between herbs well, and the topic models were difficult to capture the higher-order correlation of herbs or symptoms.

In order to obtain prescriptions which are more in line with TCM prescribing concepts and achieve prescription “generation” rather than herbal “mapping”, more researches on prescription generation have focused on the use of seq2seq structures [22–24]. Wang et al. [25] viewed TCM prescription generation as a machine translation problem. They proposed a seq2seq model to explore potential associations among symptoms and decoded such associations into prescriptions. The model designed by Liu et al. [26] learned the interaction between symptoms and herbs by simulating the correspondence between clinical consultation and prescription. And then this model used an attention mechanism that identified the primary and secondary symptoms to assist prescription generation. Undeniably, these models based on seq2seq have achieved good results. However, the current extraction of key information about symptoms is limited by the infrastructure of the seq2seq model. And the identification of some key information is not sufficient. At the same time, the existence of information that can be used in prescriptions closely related to symptoms is also ignored, which leads to generate inaccurate prescriptions. In addition, there is another problem that needs to be solved urgently. The concept of herbal preselection proposed by Li et al. [24] is a new idea to guide prescription generation. However, its herbal preselection method simply selected herbs that can treat symptoms. In the known prescription generation studies, there has never been a method to correlate herbal preselection to compatibility theory.

To address the above issues, this paper establishes new Chinese herbs guided and prescription candidates' attention depth model based on symptoms text. This model incorporates TCM background knowledge into the seq2seq structure to assist in prescription generation. The originality of our proposed method has two main components. On the one hand, this paper retains the original encoder structure for extracting critical symptoms information, and additionally uses prescription knowledge to build a branch network to guide the extraction of the symptoms information. This cross-conceptual knowledge source guidance method will be a worthwhile attempt. On the other hand, our method also incorporates the knowledge of TCM prescription compatibility. The concept of herbal preselection proposed by Li et al. [24] is expanded to a larger scope by selecting a set of prescriptions as candidate herbs. These prescriptions can treat symptoms similar to those being treated. This paper hopes that the selected candidate herbs will not only treat the symptoms but also balance the overall efficacy of the prescriptions. In conclusion, we propose a dual-branch guidance strategy combined with candidate attention model (DGSCAM) to automatically generate TCM prescriptions based on symptoms text. Firstly, in order to play the guiding role of prescription efficacy on symptoms information extraction, a dual-branch information extraction module (DIEM) is designed. This module extracts the intermediate vector of prescription efficacy, and guides the bidirectional hybrid encoder (EnGRU) to improve its own performance (Section 3.2). Secondly, we propose a task-qualified knowledge base matching module (TKMM). It guides the generation of the prescription-based herbal candidates pool based on the knowledge of TCM prescription compatibility (Section 3.3). In addition, this paper proposes a multi-dimensional complementary attention module (MCAM) to fuse the attention of the prescription-based herbal candidates pool with that of the conventional herbal library (Section 3.4). Then, to obtain prescriptions without duplicate herbs, the candidates coverage decoder (DeGPT2) is mainly built by integrating TKMM and MCAM (Section 3.5).

The main contributions of this paper are as follows:

1. We propose a novel seq2seq-based Chinese herbs guided and prescription candidates attention depth model to generate TCM prescriptions. A dual-branch guidance strategy combined with candidate attention model (DGSCAM) is designed to analyze symptoms text information and generates prescriptions that are consistent with the concept of TCM
2. For all we know, our work is the first to investigate the effect of prescriptions as herbal candidates on prescription generation. We propose a dual-branch information extraction module (DIEM), so that prescriptions can be used to guide the extraction of symptoms information. At the same time, in order to give full play to the positive effect of TCM prescription compatibility rules on the automatic generation of TCM prescriptions, we also propose a task-qualified knowledge base matching module (TKMM)
3. The feasibility of this method has been verified by experiments. Feeding symptoms text into our model generates symptomatic prescriptions with overall efficacy. The results show that our model has certain advantages over the currently popular prescription generation models. Meanwhile, the method has been recognized by professional TCM doctors

## 2. Related Work

### 2.1. Dual-Branch Network

Since many problems do not exist in isolation, they can all be solved through domain knowledge and multidimensional thinking [27, 28]. Guided by the above thinking concepts, the dual-branch network is becoming more and more momentous. Extensive works [29–32] have shown that dual-branch network can not only reduce computational cost but also have the potential to improve task performance. Chen et al. [33] proposed a dual-branch adversarial learning framework in the task of named entity recognition, where one branch learned entity domain knowledge, and the other branch captured better features, the two aspects worked together to improve the results. Chen et al. [34] combined a Bayesian network based on medical knowledge graph augmentation with a multibranch entity-aware convolutional neural network. They used the multibranch network to extract each basic information in electronic medical records (EMR) separately for comprehensive analysis. Finally, they constructed a network model for automatic diagnosis based on EMR. Deng et al. [35] used a dual-branch adversarial learning model based on keyword information to complete the Chinese text summarization task. The application of the dual-branch network not only reduced the probability of incomplete information extracted by the encoder but also served as a guide for the generation of target summaries. All the studies mentioned above have demonstrated from different perspectives that the dual-branch network can improve the learning ability of the subject task through knowledge extraction from multiple perspectives.

### 2.2. Attention Mechanism

The attention mechanism can automatically learn and calculate the contributing degree of the input data to the output data. And it can explore the regions that need to be focused in a focused manner and suppresses the interference of weakly relevant information. Bahdanau et al. [36] used an attention-like mechanism to simultaneously perform translation and alignment on machine translation task. It was the first proposal to apply the concept of attention mechanism to the field of natural language processing (NLP). Wang et al. [25] conducted a study on the generation of TCM prescriptions based on machine translation. The study used scaled dot-product attention and multihead transformation parallel computation to extract symptoms information. This approach can better explore potential correlations between symptoms. Li et al. [24] tried to use the knowledge of herbal effects to enhance the effect of TCM prescription generation. At the same time, these scholars also used multiple attention mechanisms to assist the acquisition of symptoms information. Liu et al. [26] used two kinds of attention in a study on TCM prescription generation. They were, respectively, main-subordinate symptom attention and dynamical herb attention. The first type of attention emphasizes which symptoms have a more significant impact on the disease. The second type of attention achieves the effect that the herbs treating the main symptoms were first predicted. Ruan et al. [23] also studied the attention mechanism in the generation of TCM prescriptions. They used attention mechanisms to explore the relationships between different symptoms and different herbs. These relationships included, but were not limited to, the same herbs for different symptoms, and different herbs for the same symptoms. Liu et al. [37] proposed the transfer learning model of the few-shot learning to generate TCM prescriptions. This study used multiple levels of attention during the prescription-generation phase to dig into the associations between symptoms information, medical history information, and herbs information.

## 3. Material and Method

**Figure 1:**
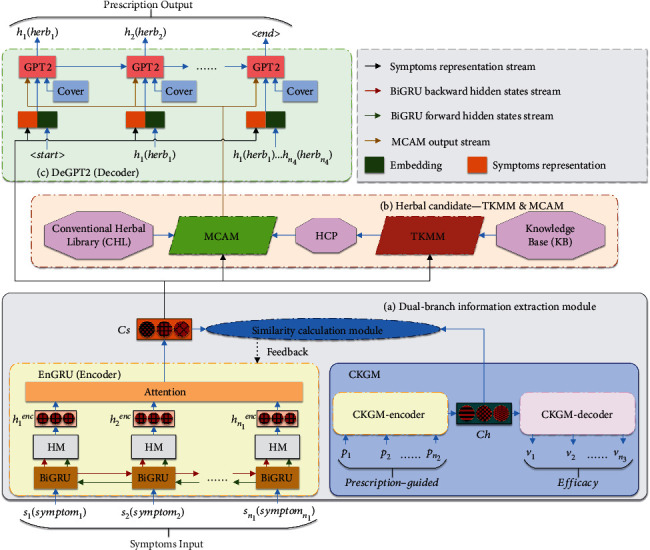
The framework of the proposed GSCCAM. (In CKGM, CKGM-encoder and CKGM-decoder are, respectively, the encoder and decoder of CKGM; *P*(*p*_1_, *p*_2_, ⋯, *p*_*n*_2__) is the prescription-guided; *V*(*v*_1_, *v*_2_, ⋯, *v*_*n*_3__) is the efficacy; *Ch* is the content representation of the prescription-guided efficacy. In EnGRU (encoder), HM is the hybrid module; *h*_*i*_^enc^ is the final hidden state of the symptoms at the *i*^th^ step. In DeGPT2 (decoder), *H*(*h*_1_, *h*_2_, ⋯, *h*_*n*_4__) is generated prescription; Cover is the new coverage mechanism.).

An overview of the dual-branch guidance strategy combined with candidate attention model (DGSCAM) proposed in this paper is shown in Figure 1. The model is based on the seq2seq structure and can well integrate TCM prescription knowledge for automatic generation of intelligent TCM prescriptions. First, in the encoding part, in order to allow the bidirectional hybrid encoder (EnGRU) to extract more accurate content representation *Cs* from the symptoms *S*(*s*_1_, *s*_2_, ⋯, *s*_*n*_1__), this paper designs a dual-branch information extraction module (DIEM). The branch of the cross-concept knowledge source guidance module (CKGM) in DIEM can assist the EnGRU branch to extract *Cs* using the potential association of prescriptions with symptoms. Second, in the decoding part, in order to consider the compatibility knowledge of TCM prescriptions, this paper proposes a task-qualified knowledge base matching module (TKMM). TKMM calculates the matching of symptoms *S* with the symptoms in the knowledge base (KB) built in this paper, and obtains the base prescriptions to form the prescription-based herbal candidates pool (HCP). Meanwhile, we propose the multidimensional complementary attention module (MCAM). MCAM combines the attention of HCP with the attention of the conventional herbal library (CHL) to improve the quality of generated prescriptions. Moreover, we improve a new coverage mechanism (Cover) to solve the problem of duplicate herbs in prescriptions *H*(*h*_1_, *h*_1_, ⋯, *h*_*n*_4__) generated by the candidates' coverage decoder (DeGPT2). We will further explain the four important components of the model in this paper: DIEM, TKMM, MCAM, and DeGPT2 in the following sections.

### 3.1. Datasets

**Figure 2:**
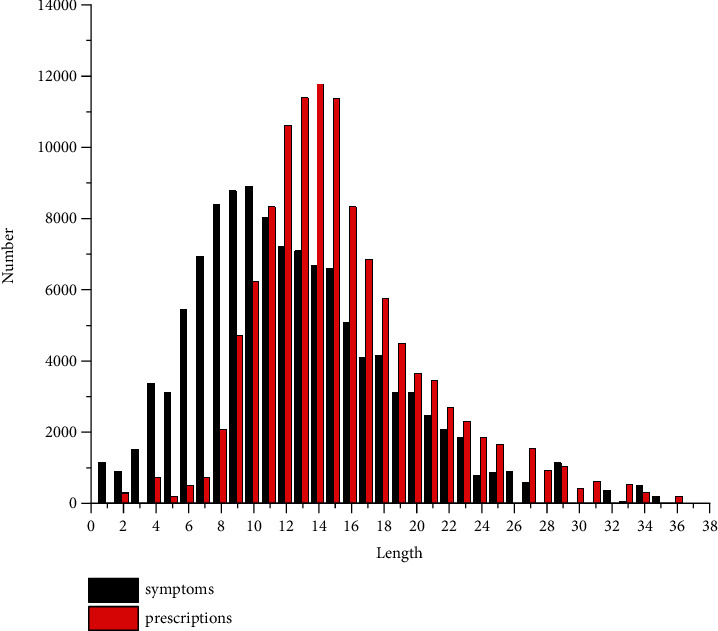
Distribution of symptoms sequence lengths and herbal quantities and their corresponding prescription quantities.

In this work, we use two datasets: the “symptoms-prescription” data pairs set (SP) and the “prescription-efficacy” data pairs set (PE). (1) There are three sources of the “symptoms-prescription” data pair set (SP), namely the public data set (CKCEST) [23], the traditional diagnostic records extracted from the ancient Chinese medical books (ACMB), and the clinical diagnosis records provided by cooperative Chinese medicine company (CCMC). Among them, the CKCEST and ACMB, respectively, contain 33,765 and 80,420 “symptoms-prescription” data pairs. CCMC contains 1,265 real clinical records in the form of “symptoms-prescription” data pairs. We clean the acquired data using TCM symptom differential diagnosis [38] and the TCM MeSH [25]. After normalized processing, we perform statistics on the resulting 115,450 “symptoms-prescription” data pairs (dataset 1), and obtain the distribution of symptoms sequence length and the distribution of herbs quantity in prescriptions. As shown in Figure 2, about 91.96% of the prescriptions contain no more than 21 symptoms and no more than 23 herbs. In order to avoid the dependence of model on too long data and lead to overfitting situations, we remove the data that do not satisfy these conditions. Ultimately, this paper obtains a dataset (SP) with 106,168 “symptoms-prescription” data pairs. To make the experimental results more robust, we randomly divide the dataset SP into two parts, 95,551 data for training (90%) and 10,617 data for testing (10%). (2) The “prescription-efficacy” data pairs set (PE) are derived from CKCEST and ACMB. The original data is standardized and normalized as above, and finally the PE containing 25,563 “prescription-efficacy” data pairs is obtained. Likewise, we divide the training set and test set in a ratio of 9 : 1.

### 3.2. Dual-Branch Multiview Information Extraction Module

#### 3.2.1. Bidirectional Hybrid Encoder

EnGRU compresses the symptoms *S* into *Cs* through the knowledge of TCM syndrome differentiation. Since there are multiple complex logical relationships among a group of symptoms, the expression of each symptom does not independently represent a certain function of the body. Therefore, when extracting *Cs* from symptoms *S*, it is necessary to comprehensively consider the relationship among the symptoms. To this end, this paper encodes the symptoms *S*(*s*_1_, *s*_2_, ⋯, *s*_*n*_1__) using the bidirectional GRU [39] defined by

(1) $$\begin{array}{c} {h_{i}^{\text{enc}}}^{\prime }=\text{BiGRU}\left(s_{i},{h_{i}^{\text{enc}}}^{\prime }\right). \end{array}$$

This can get the forward hidden states *H*_*f*_^enc^′ = {*h*_1,*f*_^enc^′, ⋯⋯, *h*_*i*,*f*_^enc^′, ⋯⋯, *h*_*n*_1_,*f*_^enc^′} and the backward hidden states *H*_*b*_^enc^′ = {*h*_1,*b*_^enc^′, ⋯⋯, *h*_*i*,*b*_^enc^′, ⋯⋯, *h*_*n*_1_,*b*_^enc^′}. Meanwhile, the hybrid module (HM) is designed to adaptively weight the hidden states in both directions. The final hidden states of the symptoms *H*^enc^ = {*h*_1_^enc^, ⋯⋯, *h*_*i*_^enc^, ⋯⋯, *h*_*n*_1__^enc^} are calculated by

(2) $$\begin{array}{c} h_{i}^{\text{enc}}=w_{i}^{s}\times {h_{i,f}^{\text{enc}}}^{\prime }+\left(1-w_{i}^{s}\right)\times {h_{i,b}^{\text{enc}}}^{\prime }. \end{array}$$

Here, the parameter *w*_*i*_^*s*^ is the weight of the influencing degree of the forward-propagating hidden states on *s*_*i*_. Its calculation method is shown in

(3) $$\begin{array}{c} w_{i}^{s}=\text{sigmoid}\left(w_{z}\left({h_{i,f}^{\text{enc}}}^{\prime },h_{i-1}^{\text{enc}},s_{i-1},\underset{1\leq j\leq i}{\sum }\left(s_{i}\times w_{j}^{s}\right)\right)+b_{z}\right). \end{array}$$

*w*_*z*_ and *b*_*z*_ are the learning parameters. The final hidden states *H*^enc^ are computed by the attention mechanism of Equation (4). Then, we obtain the content representation *Cs*_*t*_ of input symptoms *S* at the *t*^th^ step.

(4) $$\begin{array}{c} {Cs}_{t}=\overset{n_{1}}{\underset{i=1}{\sum }}\left(a_{ti}∙h_{i}^{\text{enc}}\right), \end{array}$$

where *a*_ti_ = softmax(*V*^*T*^∙tanh(*w*_*s*_∙*s*_*t*−1_^dec^ + *w*_*h*^enc^_∙*h*_*t*_^enc^)); *w*_*s*_ and *w*_*h*^enc^_ are learning parameters in *a*_ti_. *s*_*t*−1_^dec^ is the hidden state of the DeGPT2, which will be introduced in the subsequent Section 3.5.

#### 3.2.2. Cross-Concept Knowledge Source Guidance Module

**Figure 3:**
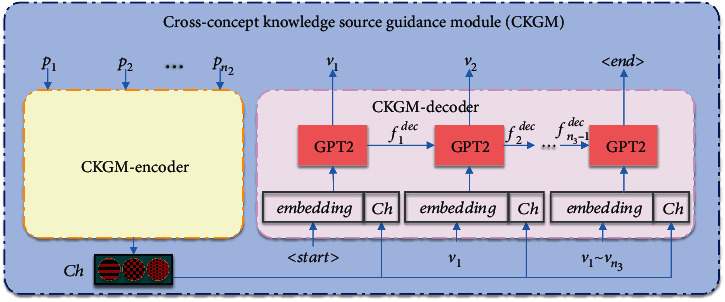
Structure chart of the cross-concept knowledge source guidance module (CKGM).

In the research on TCM prescriptions, it is generally accepted that the internal representation of symptoms can be manifested in another perspective by the efficacy of the corresponding prescription [40]. However, few studies on TCM prescription generation have used prescriptions as a knowledge source to guide the extraction of symptoms information. Guided by traditional Chinese medical knowledge [41], this paper attempts to use the efficacy *Ch* of symptoms-related prescription-guided to directly influence the extraction of symptoms content representation *Cs*. Although *Ch* does not directly act on the generation of the final target prescriptions. However, since *Ch* assists in the generation of *Cs*, it belongs to indirectly influencing the generation of the final target prescriptions. This paper hopes to obtain the more complete and accurate content representation *Cs* of symptoms *S* by exploiting the potential correlation between prescription and symptoms. To achieve this goal, we construct a cross-concept knowledge source guidance module (CKGM), as shown in Figure 3.

CKGM is guided by the theoretical knowledge of TCM on the efficacy of prescription extraction. And it constrains the network by the loss value of the generation efficacy *V*(*v*_1_, *v*_2_, ⋯, *v*_*n*_3__) and the label efficacy *V*(*v*_1_′, *v*_2_′, ⋯, *v*_*n*_5__′) of the prescription *P*(*p*_1_, *p*_2_, ⋯, *p*_*n*_2__). Eventually, the trained CKGM can extract the content representation *Ch* that can represent the efficacy of prescription *P*. The prescription encoder CKGM-encoder and the efficacy decoder CKGM-decoder of CKGM are described below, respectively.

*(1) Prescription Encoder CKGM-Encoder*. According to the theory of TCM prescription, the herbs within a prescription do not function individually, but synergistically and interactively. It is the same idea as the logical relationships among symptoms. So the encoder of CKGM uses the structure of the EnGRU as shown in Figure 1. After inputting the prescription *P* into the CKGM-encoder, we obtain the content representation *Ch*_*t*_ of input prescription *P* at the *t*^th^step.

*(2) Efficacy Decoder CKGM-Decoder*. GPT2 [42] is a character-level language model with deeper self-attention and autoregressive properties. It can take into account the generated content and predict one letter or character at a time, which is very much in line with the research idea of this paper. Therefore, this paper chooses a pretrained GPT2 model with 117 M parameters as the CKGM-decoder. The most likely prescription efficacy output *v*_*t*_ can be expressed by

(5) $$\begin{array}{c} v_{t}=\arg\;\underset{v}{\max}\;P\left(v_{t}\left|g_{t-1},\text{Ch},v_{1},v_{2},\cdots \cdots ,v_{t-1}\right.\right), \end{array}$$

where *g*_*t*−1_ = GPT2(Ch, *g*_*t*−2_, *v*_1_, ⋯⋯, *v*_*t*−2_) is the hidden state of the CKGM-decoder at the (*t* − 1) ^th^ step. After the above calculation process, the prescription efficacy *V* calculated from the prescription *P* is obtained.

#### 3.2.3. Similarity Calculation Module

For jointing the guidance information Ch of prescription, we propose the similarity calculation module (SCM). As shown in Equation (6), the semantic distance SC between Cs and Ch is calculated by the cosine similarity function as follows:

(6) $$\begin{array}{c} \text{SC}=\frac{\text{Cs}∙\text{Ch}}{\left\|\text{Cs}\right\|∙\left\|\text{Ch}\right\|}. \end{array}$$

If SC is greater than or equal to the threshold *δ* set in this paper (*δ* is set to 0.6 in this paper), then it can indicate that the Cs computed by the EnGRU can already represent the information of symptoms *S* completely and accurately. At this time, the supervision of the EnGRU by CKGM is reduced. If SC is smaller than the threshold *δ* set in this paper, it indicates that the EnGRU is not yet able to extract Cs well. And it needs to be supervised more by CKGM to achieve the expected effect.

### 3.3. Task-Qualified Knowledge Base Matching Module

TCM doctors prescribe prescriptions under the guidance of the theory of prescription compatibility [43]. At the same time, TCM doctors generally prescribe prescriptions based on their own past experiences and ancient medical books in history as references. In order to improve the rationality of the prescription generated by the model, and also to make the prescription generation based on a traceable basis, this paper proposes a task-qualified knowledge base matching module (TKMM). We expect that TKMM can construct a prescription-based herbal candidates pool (HCP) based on the pre-created knowledge base (KB) and Cs. The HCP is used to store symptom-related herbs and provide herbal candidates for subsequent prescription generation. It should be noted that some of the herbs selected in the HCP can treat diseases, and some only play an auxiliary role.

The knowledge base (KB) uses dataset 1 as the essential data. This paper calculates the content representation KB_Cs_^*i*^ of each symptom KB_*S*_^*i*^ in dataset 1, and stores the result in the data group KB_*i*_ of “symptoms-prescription-content representation”. Taking the data group as the basic unit, a D×3-dimensional matrix is formed, that is the knowledge base (KB).

To populate HCP for symptoms *S*, this paper calculates the correlation of Cs with KB_*Cs*_^*i*^. The improved similarity calculation method based on the syndrome knowledge of TCM is shown in

(7) $$\begin{array}{c} \text{Sim}=\lambda_{1}\left(1-\text{SP}\right)+\lambda_{2}\text{Nor}\left(\text{ED}\right),\\ \text{SP}\left(\text{Cs},{\text{KB}}_{\text{Cs}}^{\text{i}}\right)=\frac{\sum \text{C}\text{s}∙{\text{KB}}_{\text{Cs}}^{i}}{\sqrt{\left(\sum {\text{Cs}}^{2}\right)\cdot \left(\sum {{\text{KB}}_{\text{Cs}}^{i}}^{2}\right)}},\\ \text{ED}\left(\text{Cs},{\text{KB}}_{\text{Cs}}^{i}\right)=\sqrt{\sum {\left(\text{Cs}-{\text{KB}}_{\text{Cs}}^{i}\right)}^{2}}, \end{array}$$

Here, SP denotes the comparison method of spatial vector similarity; ED denotes the calculation method of Euclidean distance similarity; and Nor(ED) denotes the normalization of Euclidean distance. In particular, *λ*_1_ and *λ*_2_ are adjustable parameters for balancing the contributions of the two similarity methods, and in this paper, *λ*_1_ is set to 0.4 and *λ*_2_ is set to 0.6.

The similarity calculation results are sorted from smallest to largest. The prescriptions corresponding to the first *k* similar KB_Cs_^*i*^ are selected, and the herbs contained in these prescriptions are used to form the HCP.

### 3.4. Multidimensional Complementary Attention Module

**Figure 4:**
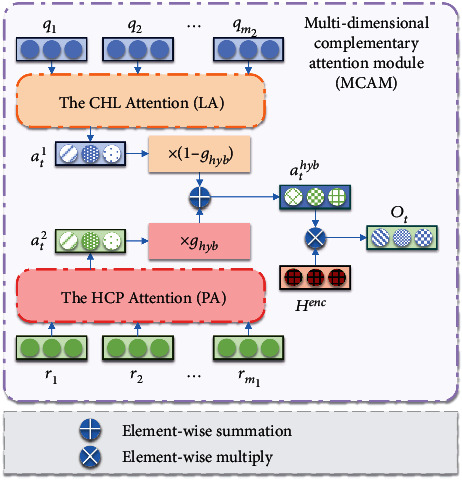
Structure chart of the multidimensional complementary attention module (MCAM). ((*r*_1_, *r*_2_, ⋯, *r*_*m*_1__) are herbs in the HCP, and (*q*_1_, *q*_2_, ⋯, *q*_*m*_2__) are herbs in the CHL.).

In order to better generate TCM prescriptions, it is not enough to have only the symptoms-related HCP but also need to be supplemented with conventional herbal library (CHL) that contains more herbs. In dataset 1, many herbs are used less frequently and are not in the current list of commonly used TCM herbs. Therefore, we select herbs that occur more than 480 times to form the CHL. For the two candidate herbal forms to complement each other, we propose a multidimensional complementary attention module (MCAM) as shown in Figure 4. The attention distribution *a*_*t*_^1^ of the herbs in the CHL is calculated by the attention of the conventional herbal library (LA). And the attention distribution *a*_*t*_^2^ of the herbs in the HCP is calculated by the attention of the prescription-based herbal candidates pool (PA). The specific implementation process is shown

(8) $$\begin{array}{c} a_{t}^{1}=\text{softmax}\left(V_{1}^{T}\cdot \tanh\left(w_{q}∙q_{i}+w_{qs}∙s_{t}^{\text{dec}}+b_{q}\right)\right),\\ a_{t}^{2}=\text{softmax}\left(V_{2}^{T}\cdot \tanh\left(w_{r}∙r_{j}+w_{rs}∙s_{t}^{\text{dec}}+b_{r}\right)\right), \end{array}$$

Here, *q*_*i*_ is the *i*_th_ herb in the CHL; *r*_*j*_ is the *j*_th_ herb in the HCP; *s*_*t*_^dec^ is the hidden state at *t*^th^ step of the DeGPT2; and *V*_1_^*T*^, *V*_2_^*T*^, *w*_*q*_, *w*_*r*_, *w*_*qs*_, *w*_*rs*_, *b*_*q*_, and *b*_*r*_ are the learning parameters.

In order to combine the two types of attention, we need an adaptive parameter *g*_hyb_ as shown in

(9) $$\begin{array}{c} g_{hyb}=\text{sigmoid}\left(w_{\text{hyb}}∙\left[s_{t-1}^{\text{dec}},\underset{i}{\sum }\left(q_{i}∙a_{ti}^{2}\right),h_{t-1}\right]\right), \end{array}$$

where *w*_hyb_ is a learning parameter. *h*_*t*−1_ and ∑_*i*_(*q*_*i*_∙*a*_*ti*_^2^), respectively, emphasize the LA and the PA, so *g*_*hyb*_ can represent two kinds of attention. Meanwhile, since ∑_*i*_(*q*_*i*_∙*a*_*ti*_^2^) emphasizes the PA, so *g*_hyb_ pays more attention to the PA. After the adaptive parameter *g*_hyb_ is obtained, the complementary attention distribution *a*_ti_^hyb^ and the complementary attention context vector.

*O*_*t*_ are calculated by

(10) $$\begin{array}{c} a_{\text{ti}}^{\text{hyb}}=g_{\text{hyb}}∙a_{\text{ti}}^{2}+\left(1-g_{\text{hyb}}\right)∙a_{\text{ti}}^{1}, \end{array}$$

(11) $$\begin{array}{c} O_{t}=\underset{i}{\sum }a_{ti}^{\text{hyb}}∙h_{i}^{\text{enc}}. \end{array}$$

Here, *h*_*i*_^enc^ is the final hidden state of the EnGRU at the *i*^th^ step.

### 3.5. Candidates Coverage Decoder

The candidates coverage decoder (DeGPT2) is based on GPT2. To bring the generated prescriptions closer to the prescribing level of TCM doctors, DeGPT2 fuses the new coverage mechanism (Cover) with the multidimensional complementary attention module (MCAM) to generate targeted prescriptions *H*(*h*_1_, *h*_1_, ⋯, *h*_*n*_4__) with the content representation Cs of symptoms. The task of generating TCM prescriptions requires that there are no duplicate herbs in the final generated prescriptions. Inspired by Li et al. [44], this paper encourages the model to generate new herbs and suppress existing ones through the Cover. It can reduce the probability of duplicating herbs in the generated prescriptions and improve the recall rate. The process of implementing the Cover is shown as

(12) $$\begin{array}{c} a_{t}^{3}=\text{softmax}\left(V_{3}^{T}∙\tanh\left(w_{h^{\text{enc}}}∙h_{k}^{\text{enc}}+w_{m}∙s_{t}^{dec}+w_{\mathrm{cov}}∙\left[h_{1},\cdots \cdots ,h_{t}\right]+b_{r}\right)\right), \end{array}$$

(13) $$\begin{array}{c} c_{t}^{3}=\underset{k}{\sum }a_{\text{tk}}^{3}∙h_{k}^{\text{enc}}, \end{array}$$

where *h*_*k*_^enc^ is the final hidden state of the EnGRU at the *k*^th^ step; *h*_1_, ⋯⋯, *h*_*t*_ are the t herbs that have been generated; *a*_*t*_^3^ is the coverage vector; and *s*_*t*_^dec^ is the hidden state of the DeGPT2 at the *t*_*th*_ step, which calculated by

(14) $$\begin{array}{c} s_{t}^{\text{dec}}=GPT2\left(s_{t-1}^{\text{dec}},h_{1},\cdots \cdots ,h_{t},O_{t},Cs,c_{t}^{3}\right). \end{array}$$

When the DeGPT2 generates a prescription, there are two sources of herbs that make up the prescription. One is copied from two candidate herbs, and the attention distribution can be regarded as the replication probability of each candidate herb at *t*^th^ step. The other is generated by the decoder. The herb *h*_*t*_ predicted by our DeGPT2 at *t*^th^ step is obtained by combining the vocabulary generation probability *P*_vocab_ with the candidate herbs replication probability *P*_candidate_. The calculation method of the two probabilities is shown as

(15) $$\begin{array}{c} P_{\text{vocab}}=\text{softmax}\left(V_{\text{vocab}}∙\left[s_{t-1}^{\text{dec}},\text{Cs}\right]+b_{\text{vocab}}\right),\\ P_{\text{candidate}}=\underset{j}{\sum }a_{tj}^{\text{hyb}}, \end{array}$$

where *V*_vocab_ and *b*_vocab_ are the learning parameters. The two probabilities have different sources and require the weight value *p*_gen_ ∈ [0, 1] shown in Equation (16) to control the final result:

(16) $$\begin{array}{c} p_{\text{gen}}=\text{sigmoid}\left(w_{s}∙s_{t}^{\text{dec}}+w_{p}∙h_{t-1}+w_{Cs}∙Cs+b_{\text{gen}}\right). \end{array}$$

Here, *w*_*s*_, *w*_*p*_, *w*_Cs_, and *b*_gen_ are all learning parameters.

The final probability of *h*_*t*_ is obtained by combining *P*_vocab_ and *P*_candidate_ by Equation (17) as follows:

(17) $$\begin{array}{c} P\left(V_{t}\right)=p_{\text{gen}}∙P_{\text{vocab}}+\left(1-p_{\text{gen}}\right)∙P_{\text{candidate}}. \end{array}$$

### 3.6. Loss Function

In the task of extracting Ch (Section 3.2.2), we propose a multilevel loss function for training CKGM as shown as

(18) $$\begin{array}{c} {\text{LOSS}}_{\text{CKGM}}=-\varphi_{1}\underset{t}{\sum }\left(\frac{1}{2}\left(\frac{{q_{v}}^{\prime }}{M_{1}}+{v_{t}}^{\prime }\right)\right)\log\left(v_{t}\right)+\varphi_{2}\left(-\underset{n_{2}}{\sum }\left(\underset{v_{t}}{\sum }\left({v_{ti}}^{\prime }\log\left(v_{ti}\right)\right)\right)\right). \end{array}$$

Among them, *v*_*t*_′ is the label efficacy of the prescription *P*; *v*_*t*_ is the generation efficacy of the prescription *P*; *v*_*ti*_′ is the component of the label efficacy; *v*_*ti*_ is the component of the generation efficacy; *q*_*v*_′ is a vector with dimension equal to the number of all existing efficacies, set to 1 at the “label efficacy” position and 0 at the rest; *M*_1_ is the number of efficacies in the label efficacy; *φ*_1_ and *φ*_2_ are the parameters automatically learned by the model.

*LOSS*_*CKGM*_ is composed of the soft loss function [44] and the traditional loss function. This paper uses the soft loss function because there is no strict semantic order among multiple efficacies of TCM prescriptions. This paper uses the traditional loss function because each efficacy is a phrase or sentence with independent semantics, and there needs to be a strict semantic order within the efficacies.

Inspired by Verboven et al. [45], this paper designs the loss function LOOS_final_ as shown in Equation (19) to train DGSCAM.

(19) $$\begin{array}{c} {\text{LOOS}}_{\text{final}}=-\psi_{1}\underset{t}{\sum }\left(\frac{1}{2}\left(\frac{{q_{h}}^{\prime }}{M_{2}}+{h_{t}}^{\prime }\right)\right)\log\left(h_{t}\right)+\psi_{2}{\text{LOSS}}_{\text{CKGM}}, \end{array}$$

where *h*_*t*_ is the herb generated at *t*^th^ step of the DeGPT2; *h*_*t*_′ is the labeled herbs at *t*^th^ step; *q*_*h*_′ is the one-hot code of the labeled prescription; *M*_2_ is the number of herbs in the labeled prescription. *ψ*_1_ and *ψ*_2_ are the weights for the two tasks, which are computed dynamically by our model.

The loss function LOOS_final_ is composed of the soft loss function [44] and the multilevel loss function LOSS_CKGM_. It can well regulate the training process of DGSCAM. This paper uses the soft loss function because our task does not require strict word order for the herbs in generated prescriptions.

## 4. Results and Discussion

### 4.1. Implementation Details

All experiments are conducted on a workstation with Ubuntu 18.04 LTS, Intel(R) Xeon(R) W-2102 CPU and NVIDIA TITAN XP GPU. This paper implemented GSCCAM based on PyTorch. Our model sets the embedding size of Chinese characters in symptoms and prescription markers to 512, and the hidden states size of bidirectional GRU and GPT2 to 512 as well. We use the Adam optimizer (base learning rate is 0.001, beta_1_ is 0.9, beta_2_ is 0.999, epsilon is 1e-6, decay is 0, correct_bias is True), set the epoch number to 500 and the batch size to 64. During the training process, the model with the lowest total loss is saved for validation on the test set.

### 4.2. Evaluation Metrics

#### 4.2.1. Automatic Quantitative Evaluation Metrics

In order to automatically evaluate the performance of our model, this paper selects precision, recall, and F1 score as automatic quantitative evaluation metrics, which are commonly used in studies on TCM prescription generation. For all “symptoms-prescription” data pairs (*S*, *P*^label^) in the test dataset, they are defined as

(20) $$\begin{array}{c} \text{Precision}=\frac{\left|H\cap P^{\text{label}}\right|}{\left|H\right|},\\ \text{Recall}=\frac{\left|H\cap P^{\text{label}}\right|}{\left|P^{\text{label}}\right|},\\ F1-\text{score}=\frac{2\left|H\cap P^{\text{label}}\right|}{\left|H\right|+\left|P^{\text{label}}\right|}, \end{array}$$

where *H* represents the prescription sequence generated by the model GSCCAM. *P*^label^ represents the “prescription” in the “symptoms-prescription” data pair, that is, the label prescription sequence.

#### 4.2.2. Human Qualitative Evaluation Metrics

Since our model takes into account the compatibility idea of TCM prescriptions in the final generation of prescriptions, the labeled prescriptions within the dataset are not necessarily the only choice for treating symptoms. However, the above automatic quantitative evaluation metrics can only calculate the quality of the model based on labeled prescriptions, and do not take into account the diversity of herbs and the concept of compatibility of prescriptions. So these metrics can only represent part of the performance of our model. Therefore, to evaluate our model more comprehensively, we invited four TCM doctors from Shanxi University of Traditional Chinese Medicine to independently perform the manual qualitative evaluation of the prescriptions generated by our model. Each doctor evaluates in terms of herbal effectiveness (HE) and herbal compatibility (HC) [26], both of which range from (0, 5), and higher scores indicate better effectiveness or compatibility.

### 4.3. Baseline Models

This paper chooses the following baseline models for comparison.

1. KDHR [18]: a graph convolution model for multilayer information fusion with herbal attributes as auxiliary knowledge
2. PTM [21]: the topic model that can describe the prescription generation process according to the theory of TCM, and it used the connection between herbs and symptoms and the respective internal connections between the two as the implementation of the TCM domain knowledge guidance model
3. Coverage-Soft-Model [44]: it is a seq2seq model with a decoder having a coverage mechanism and a soft loss function
4. Herb-Know [24]: a seq2seq model that incorporated external knowledge of herbs
5. TPGen [23]: a seq2seq model using lite Transformer and bidirectional gated recurrent unit combined with TCM clinical knowledge

### 4.4. Contrast Experiments

#### 4.4.1. Comparative Results Using Automatic Quantitative Evaluation Metrics

To verify the performance of our model, we compare the performance differences between the baseline models in Section 4.3 and our model using the test set data.  Table 1 shows the performance comparison of our proposed GSCCAM with several other baseline models in terms of precision, recall, and F1 score. In the comparison, it is found that our GSCCAM significantly outperforms all other methods in the task of TCM prescription generation. As shown in Table 1, GSCCAM achieves 37.39% precision and 25.04% recall, and also achieves an excellent F1 score which is higher than the best baseline model TPGen by 4.81%. KDHR, PTM, and Coverage-Soft-Model all achieve relatively small precision and recall. In a sense, the multilabel classification idea of KDHR is difficult to grasp the compatibility among herbs. The topic model of PTM cannot incorporate the whole knowledge into the model when considering the prior knowledge of TCM. And Coverage-Soft-Model is limited to the coverage mechanism and soft loss function to solve the problem of duplication and weak order of herbs, which is less helpful to the target task. GSCCAM has improved these aspects and achieved the overall effect of “1 + 1 + 1 > 3” when compared with the above three models. Clearly, both Herb-Know and TPGen achieve good results on our test set. Through analysis, we believe that this is because these two models are better at capturing the relationship between symptoms and herbs than general deep learning methods.

#### 4.4.2. Comparative Results Using Human Qualitative Evaluation Metrics

Since it takes a lot of time and energy for doctors to perform human qualitative evaluation, this paper chooses to randomly sample 500 data from the test set for this work. As shown in Table 2, our GSCCAM outperforms other baseline models on both metrics, obtaining an average HE value of 4.15 and an average HC value of 3.55. This situation indicates that our model can generate effective herbs and the herbs basically conform to the principles of TCM compatibility. Both HE and HC scores of KDHR and Coverage-Soft-Model do not exceed 2.5, and the distribution of their total values is centered between [3.2, 4]. It indicates that the multilabel classification idea of KDHR does not fit well with the compatibility principles of TCM prescriptions. Meanwhile, Coverage-Soft-Model is difficult to obtain prescriptions that conform to TCM ideas because it does not focus specifically on TCM prior knowledge. It is difficult for PTM as a topic model to capture higher-order correlations of herbs or symptoms, so it can only achieve an average HE value of 2.85 and an average HC value of 1.775. Although TPGen and Herb-Know have good effects, they only consider the influence of external knowledge of TCM on the model and ignore the positive effect of general text on the model. In comparison, our model is highly approved by 4 doctors. And it is approximately 40.0% higher than the baseline model TPGen, which is the second-approved baseline model by doctors. This fully demonstrates the superiority of our GSCCAM in the prescription generation task. At the same time, our GSCCAM obtained almost unanimously optimistic results, with only the 15.81% fluctuation compared with the evaluation results of other baseline models. This indicates that our model has better stability and robustness in the field of specialized TCM.

**Figure 5:**
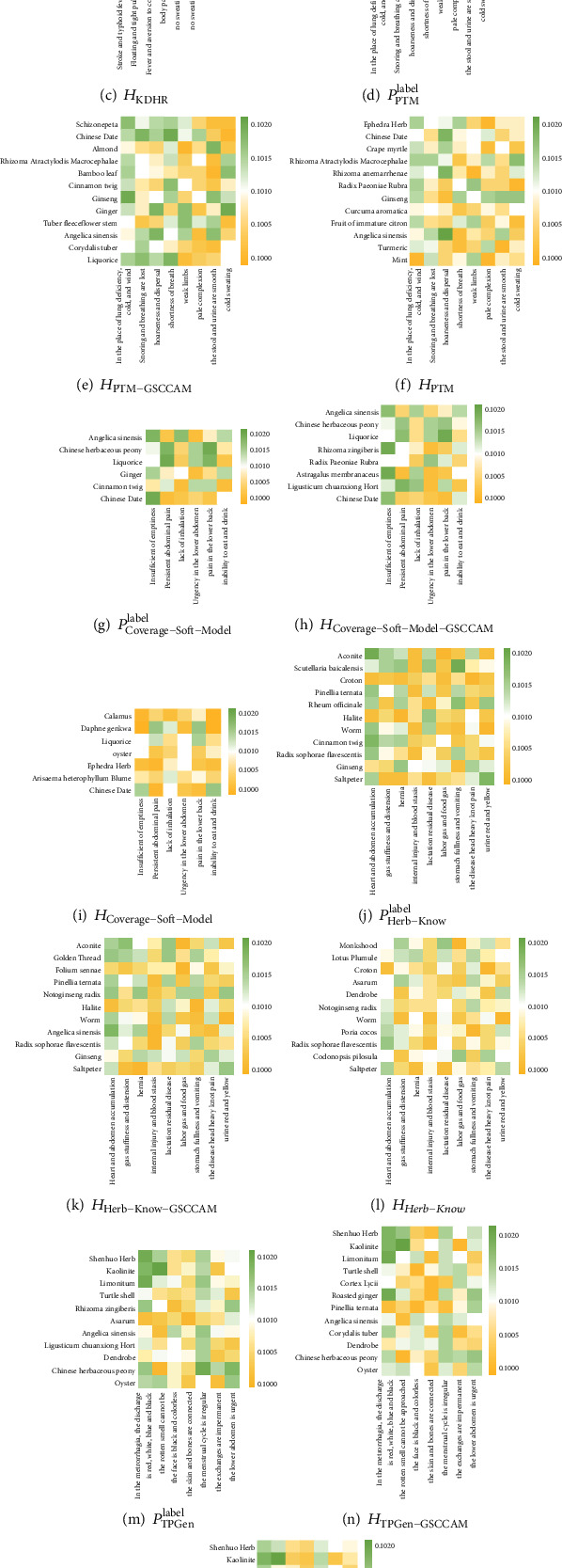
Thermodynamic comparison chart of the symptoms-herbs relationship between the baseline models and our model (GSCCAM).

Considering that there may be inconsistencies in doctors' recognition of multiple prescriptions generated by the same model. To avoid the influence of the volatility of doctors' subjective judgments on the performance evaluation of the baseline models, we take out the symptoms *S*_*i*_ corresponding to the prescription result *H*_*i*_ with the highest overall evaluation of each baseline model. Putting *S*_*i*_ into our GSCCAM to generate prescription *H*_*i*−GSCCAM_. By plotting a thermodynamic chart of the symptoms-herbs relationship of *H*_*i*_, *H*_*i*−GSCCAM_, and *S*_*i*_-labeled prescription *P*_*i*_^label^, the performance of our model versus the baseline models is more intuitively shown. As shown in Figure 5, the area in the *i*^th^ row and *j*^th^ column represents the relationship between the *i*^th^ herb and the *j*^th^ symptom. Green represents correlation, yellow represents irrelevance, and the darker color represents the heavier degree. It is easy to see from the visualization that our model can produce prescription results that are closer to the ground truth when compared to all baseline models. This shows that our model can still take into account the relationship between symptoms and herbs while considering the external knowledge of TCM herbs. And our model can balance the external knowledge of TCM herbs and the compatibility principles of TCM, and satisfactorily complete the task of prescription generation.

### 4.5. Ablation Experiments

In this section, to further evaluate the effectiveness of the proposed cross-concept knowledge source guidance module (CKGM), task-qualified knowledge base matching module (TKMM), multidimensional complementary attention module (MCAM), we conduct several ablation experiments. Under the same training settings and the same dataset, we separately verify the performance of the modules mentioned in GSCCAM and evaluate them according to the above automatic quantitative evaluation metrics.

#### 4.5.1. Double-Branch Task Analysis

To verify the effectiveness of the CKGM branch network for the dual-branch task in the proposed method, this paper chooses the base model (BM) without the addition of CKGM to compare with our model. Meanwhile, to illustrate the applicability of the decoder structure in CKGM, this paper adjusts it to the decoder structure of GSCCAM for ablation experiments. As shown in Table 3, the BM (EnGRU, DeGPT2) without the addition of CKGM produces the worst results (precision is 23.38%, recall is 17.65%, and F1 score is 20.11%). Compared to the BM (EnGRU and DeGPT2), the BM (EnGRU and DeGPT2) + CKGM (CKGM − encoder and DeGPT2) still yields unsatisfactory results, although the values of precision, recall, and F1 score are improved. We analyze that the reason for this phenomenon is that the new coverage mechanism in the DeGPT2 does not have a positive effect on the task of prescription extraction efficacy, but rather has a negative impact. In contrast, we find that adding CKGM which has CKGM-decoder as a decoder in the base model is the most effective. It achieves the precision, recall, and F1 scores of 37.39%, 25.04%, and 29.99% in the prescription generation task, respectively. By using the CKGM included the CKGM-decoder decoder to fully exploit the efficacy of prescriptions, the base model is conceptually aided in extracting the intermediate vectors of symptoms with TCM prescriptions knowledge. Therefore, we believe that using CKGM to guide the base model to extract the intermediate information of symptoms is effective.

#### 4.5.2. Task-Qualified Knowledge Base Matching Module Analysis

**Figure 6:**
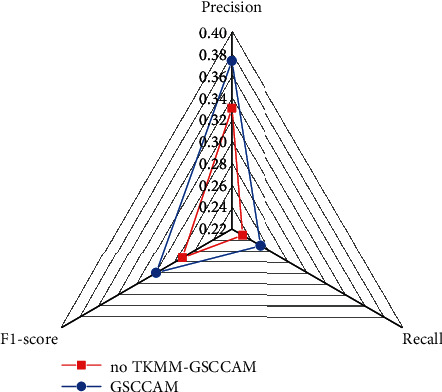
Performance comparison of the models with and without TKMM.

To demonstrate the advantages of the TKMM, this paper compares the performance of our proposed GSCCAM with the model without TKMM. For simplicity, this paper only validates the effectiveness of TKMM on the basis of the model with the addition of the most effective dual-branch structure. The experimental results are shown in Figure 6, which shows that the automatic quantitative evaluation metrics of the model are significantly reduced after removing TKMM. In noTKMM-GSCCAM, the precision, recall, and F1 score are 33.06%, 23.15%, and 27.23%, respectively, which are 4.33%, 1.89%, and 2.76% lower than those of GSCCAM, respectively. This means that TKMM can establish the HCP that is closer to the target task by screening the prescriptions with similar symptoms to be treated. The HCP can provide guidance on the direction of herbs selection for the generation of the final prescription, and help the model to come up with prescriptions that are more characteristic of TCM diagnostic thinking.

For the setting of the hyperparameters *λ*_1_ and *λ*_2_, this paper sets the different hyperparameter values by the traditional manual search method, so as to determine the best solution of the two similarity comparison methods. Since the hyperparameters *λ*_1_ and *λ*_2_ are synergistic, the basic condition of “*λ*_1_ + *λ*_2_ = 1.0” needs to be satisfied. Therefore, this paper sets the values of *λ*_1_ to (0.0, 1.0), respectively, with 0.1 as the unit of change each time; *λ*_2_ corresponds to changing its value. As shown in Table 4, we find that the model performs the best overall when *λ*_1_ and *λ*_2_ are 0.4 and 0.6, respectively. We believe that this is due to the better sensitivity of the Euclidean distance similarity calculation method and the advantage of the spatial vector similarity calculation method that does not need to be normalized. And the optimal results are obtained by combining the advantages of each. Therefore, this paper uses 0.4 and 0.6 as the optimal values of *λ*_1_ and *λ*_2_.

In addition, in order to evaluate the value of the number of candidate prescriptions *k* selected from the KB to achieve the best performance of our model, this paper uses the best values of *λ*_1_ and *λ*_2_. While this paper uses the traditional manual search method to set the value of *k* to [5, 35] with 5 as the unit of variation each time. The experimental results are shown in Table 5. When *k* is taken as 20, our model has the best performance. After analysis, we conclude that when *k* is taken as less than 20, the number of herbs in the HCP is too small, which limits the range of model selection. When *k* is taken as greater than 20, the number of herbs in the HCP is too large, which lose the significance of building a targeted set of candidates herbs. Therefore, our model selects 20 as the optimal value of *k*.

#### 4.5.3. Multidimensional Complementary Attention Module Analysis

In order to demonstrate that the two candidate herb sets of the HCP and the CHL can promote each other during the model training process, this paper conducts ablation experiments on the multidimensional complementary attention module (MCAM). This paper removes the HCP and the CHL, respectively, and observes the performance changes of the no-candidate herbal set, the single-candidate herbal set and the double-candidate herbal set. The results are shown in Table 6. Without any candidate herb set, the precision, recall, and F1 score can only reach 31.55%, 20.05%, and 24.52%, respectively. This means that without any candidates, the model can only autonomously generate the herbs that make up the prescription based on the experience obtained during training. Without the guidance of any external thinking of TCM, the effect is bound to be unsatisfactory. In the case of retaining only the CHL as candidates, the precision, recall, and F1 scores are 32.92%, 22.01%, and 26.38%, respectively. In the case of retaining only the HCP as candidates, the precision, recall, and F1 scores are 35.36%, 44.39%, and 28.87%, respectively. Judging from the situation of these two single-candidate herbal sets, it is possible to obtain better experimental results by properly guiding the model with the correct knowledge of TCM. Moreover, since the HCP contains targeted candidate herbs, it is closer to the main treatment thinking of the target task than the general high-frequency herbs in the CHL. Therefore, adding the HCP is more helpful for the model to generate prescriptions according to the TCM ideology. We can also see from the data in Table 6 that only-HCP-GSCCAM has 2.44% higher precision, 2.38% higher recall, and 2.49% higher F1 score than only-CHL-GSCCAM. When both herbal candidate sets are used in the model, the experimental results show significant improvements in precision, recall, and F1 score. This further illustrates that the double-candidate herbal set can complement strengths each other, and our proposed MCAM module can achieve this complementary operation.

### 4.6. Case Analysis

In order to better demonstrate the feasibility and reliability of our model in practical applications, this paper selects two groups of symptoms and compares the similarity between prescriptions generated by our model, prescriptions generated by the baseline models, and label prescriptions, respectively. And at the same time, the human qualitative evaluation is performed by professional doctors. The details are shown in Table 7. After comprehensively considering the performance of each model, the following conclusions are drawn.

For case 1, the similarity between the prescription predicted by KDHR and the label is very low, and only *Photinia serratifolia* is overlapped with the label content. Moreover, KDHR tends to predict high-frequency common herbs, such as *Houpoea officinalis*, *Panax notoginseng*, and *Ziziphus jujuba*. The number of generated herbs is far more than the actual required number. And the compatibility among herbs is very poor, almost irrelevant to the compatibility concept followed by TCM. PTM, as a topic model, likes to predict herbs with similar efficacy, such as *Zingiber* and *Roasted zingiber* and *Coptis chinensis* and *Phellodendron*. Meanwhile, although PTM considers the external knowledge of TCM, it is far from integrating the theoretical knowledge of TCM prescribing, and the predicted prescription also has few herbs that matched the label. In the prescription generated by Coverage-Soft-Model, only *Photinia serratifolia* is used to treat symptoms. Some of the herbs (e.g., *Mylabris*) not only do not play a curative role but even contradict the pathological nature of the symptoms. It may aggravate the condition of patient or even affect his life and health. Although Herb-Know can generate more reasonable prescriptions, the herbs *Photinia serratifolia*, *Nepeta cataria*, *Roasted zingiber*, and *Corydalis decumbens,* those are useful for the main symptoms account for less than 26.67% of the total herbs. And the rest of the herbs are mainly used to alleviate the subordinate symptoms. This shows that although Herb-Know can combine the knowledge of symptoms and herbs, its learning ability is limited. And it cannot better reflect the theoretical thinking of TCM prescribing. Three herbs (*Photinia serratifolia*, *Scutellaria baicalensis georgi*, and *Angelica sinensis*) are prescribed by TPGen that overlapped with the label. Most of the remaining herbs are mild tonics that could not be used to treat symptoms, but would not harm the patient's health. Our GSCCAM yields prescription that is roughly close to the labeled prescription. At the same time, our model predicts that *Photinia serratifolia*, *Nepeta cataria*, *Saposhnikovia divaricata* and *Zingiber* all target the “deficient wind and wind evil”, and the main function of these herbs is to dispel wind. Although they are not exactly the same as the *Photinia serratifolia*, *Dried zingiber* and *Ephedra* used in the labeled prescription, they are the more reasonable combination than the labeled prescription. *Angelica sinensis*, *dried Rehmannia glutinosa*, and *Angelica dahurica* are not treating the main symptoms, but they can be used to nourish the blood, invigorate the blood and calm the wind, and relieve the condition. As for other herbs predicted by GSCCAM, such as *Cinnamomi Ramulus*, *Scutellaria baicalensis georgi*, and *Glycyrrhiza* can be used to lower ascending and floating Qi and clear heat; *Asarum* and *Zanthoxylum ailanthoides* can be used to tonify the Yang energy of the spleen and stomach; and *Panax ginseng* can nourish Qi and support correctness.

For case 2, KDHR generates far more herbs than the number of herbs in the label, but it generates prescription that is not appropriate for treating this group of symptoms. This shows that KDHR pays great attention to the influence of herbal properties on the generated prescriptions, but ignores the connections among herbs. Therefore, in the prescriptions generated by KDHR, there are *Mentha*, *Chrysanthemum*, and other herbs those are used only for better eyesight. Prescriptions generated by PTM can be marginally effective in treating the symptoms. For example, *Cinnamomum cassia* can treat pain of the ribs under the armpit caused by the invasion of cold evil. However, because PTM has difficulty capturing higher-order correlations between herbs and symptoms, herbs with very similar efficacy (*Dried zingiber* and *Zingiber*) appear in the prescriptions it generated. *Areca catechu*, *Zingiber*, and *Cinnamomum cassia* generated by Coverage-Soft-Model all have a certain effect on the treatment of symptoms. However, *Curcuma* and *Syzygium aromaticum* are herbs that cannot be used together in the compatibility of TCM prescriptions, so this prescription has great side effects on the treatment of symptoms. Herb-Know generates herbs those can have a positive effect on treating symptoms. For example, the combination of *Astragalus membranaceus* and *Eucommia ulmoides* can nourish the liver and benefit Qi. However, there are still some herbs (*Lycium barbarum*, *Dendrobium*, etc.) in the prescription those can only achieve the most basic auxiliary functions for the treatment of symptoms. The similarity between the prescription generated by TPGen and the label prescription is relatively high. But the main feature of TPGen is the improvement of the model structure, which ignores the influence of TCM knowledge on prescription generation. So it generates herbs those are mild but useless for treating symptoms. The coincidence rate between the herbs predicted by our GSCCAM and the herbs in the label prescription is as high as 66.67%, and the remaining herbs not in the label are from herbal candidates. This group of symptoms in case 2 is mainly liver deficiency and cold invasion. In terms of the efficacy in treating symptoms, the herbs generated by our model have clear effects and reasonable collocation. For example, *Aconitum carmichaeli* can increase Yang Qi, dispel cold and relieve pain; *Zingiber* and *Astragalus membranaceus* can treat weakness and cold invasion; and *Pericarpium Citri reticulata* and *Evodia rutaecarpa* can nourish Yang Qi. From the overall performance of prescription, the prescription generated by our model is more reasonable than those generated by other baseline models.

Overall, our model does not just treat symptoms based on their correspondence with herbs. But it is influenced by the idea of potential compatibility in the prescription-based herbal candidates, analyzes and differentiates symptoms as professional TCM doctors do. And then it predicts appropriate herbs based on traditional prescription compatibility knowledge, ultimately forming a prescription with holistic, comprehensive, and correlative properties. It can also be seen through the human qualitative evaluation of doctors that our GSCCAM outperforms other baseline models. In some cases, the prescriptions predicted by our model are more reasonable than the labeled prescriptions.

## 5. Conclusion and Future Work

In this paper, we propose a novel Chinese herb guided and prescription candidates attention depth model based on symptoms text, which can guide the automatic generation of prescriptions based on theoretical knowledge of TCM. In the process of generating TCM prescriptions, our model can provide prescription-based herbal candidates targeted for treating symptoms according to the idea of prescription compatibility. To our knowledge, which is the first such attempt in artificial intelligence generation of TCM prescriptions, our model uses the DIEM to extract symptom-related information from prescriptions corresponding to symptoms. And, in order to better reflect the compatibility idea of TCM prescriptions, we add the TKMM in this model. We combine public and clinical datasets in the training and testing process of the model. In the experiments, we comprehensively analyze the performance of our GSCCAM through automatic quantitative evaluation metrics such as precision, recall, and F1 score and human qualitative evaluation metrics of professional TCM doctors. Ultimately, our model achieves 37.39% precision, 25.04% recall, and 29.99% F1 score, and achieves an average doctor score of 7.7 out of 10. The experimental results show that our model has achieved excellent automatic evaluation effect values, and has been highly recognized by TCM professional doctors. We believe that the research of this paper is full of prospective significance. This is not only because our study is based on the computation of a large amount of data but also because it is very consistent with the theoretical knowledge of traditional Chinese medicine. The research perspective of this paper breaks the shackles of the original fixed thinking in the study of combining TCM prescribing science with computer technology. It moves the research in this direction toward a more cutting-edge direction. We hope that this work can lay a foundation for future research on TCM prescription generation and attract more researchers to focus on the combination of TCM prescription and artificial intelligence.

In future work, we plan to study the association among herbs, the evolution pattern of symptoms, and herbal dosage and other information related to prescription. At the same time, we hope to introduce external knowledge of TCM such as syndrome element identification and syndrome guidance into the model. In addition, we will continue to collect more clinical data to further improve the model performance. The combination of TCM and computer technology is a very promising research. Given the serious and responsible attitude towards scientific research and the idea of seeking realism and stability, we will continue to increase our investment in this research and steadily explore the future planned work very fully, and make more and better down-to-earth achievements for this research direction.

## Figures and Tables

**Table 1:** Performance comparison of the baseline models with our model (GSCCAM) for automatic quantitative evaluation.

Models	Precision	Recall	F1-score
KDHR [18]	0.2041	0.1473	0.1711
PTM [21]	0.1982	0.1536	0.1731
Coverage-Soft-Model [44]	0.1844	0.1073	0.1357
Herb-Know [24]	0.3082	0.2101	0.2499
TPGen [23]	0.2794	0.2291	0.2518
GSCCAM	**0.3739**	**0.2504**	**0.2999**

**Table 2:** Performance comparison of the baseline models and our model (GSCCAM) by four doctors for human qualitative evaluation.

Models	Doctor1			Doctor2			Doctor3			Doctor4	
	HE	HC	Total	HE	HC	Total	HE	HC	Total	HE	HC	Total
KDHR [18]	2.2	1.5	3.7	2.3	1.1	3.4	2.0	1.2	3.2	2.4	1.6	4.0
PTM [21]	2.9	1.7	4.6	2.8	1.9	4.7	2.6	1.5	4.1	3.1	2.0	5.1
Coverage-Soft-Model [44]	1.9	1.5	3.4	2.2	1.4	3.6	2.0	1.3	3.3	2.5	1.5	4.0
Herb-Know [24]	3.5	1.8	5.3	3.1	2.3	5.4	2.9	1.9	4.8	3.8	2.2	6.0
TPGen [23]	3.7	1.8	5.5	3.2	2.2	5.4	2.9	2.1	5.0	3.6	2.5	6.1
GSCCAM	**4.3**	**3.5**	**7.8**	**4.2**	**3.4**	**7.6**	**3.9**	**3.6**	**7.5**	**4.2**	**3.7**	**7.9**

**Table 3:** Performance comparison of the model of no branch network or the model of decoder of replacing branching network and our model (GSCCAM).

Models	Precision	Recall	F1-score
BM (EnGRU,DeGPT2)	0.2338	0.1765	0.2011
BM (EnGRU, DeGPT2) + CKGM (CKGM − encoder, DeGPT2)	0.3244	0.2281	0.2679
BM (EnGRU, DeGPT2)+ CKGM (CKGM-encoder, CKGM-decoder) (ours)	**0.3739**	**0.2504**	**0.2999**

**Table 4:** Performance comparison of different combinations of hyperparameters in TKMM.

(*λ*1,*λ*2)	Precision	Recall	F1-score
(0.0,1.0)	0.3396	0.2065	0.2568
(0.1,0.9)	0.3487	0.2298	0.2770
(0.2,0.8)	0.3521	0.2330	0.2804
(0.3,0.7)	0.3653	**0.2509**	0.2975
(0.4,0.6)	**0.3739**	0.2504	**0.2999**
(0.5,0.5)	0.3711	0.2485	0.2977
(0.6,0.4)	0.3662	0.2409	0.2906
(0.7,0.3)	0.3619	0.2337	0.2840
(0.8,0.2)	0.3550	0.2291	0.2785
(0.9,0.1)	0.3498	0.2134	0.2651
(1.0,0.0)	0.3402	0.1968	0.2493

**Table 5:** Performance comparison of different number of candidate prescriptions in TKMM.

*k*	Precision	Recall	F1-score
5	0.3527	0.2396	0.2854
10	0.3571	0.2402	0.2872
15	0.3685	0.2468	0.2956
20	**0.3739**	**0.2504**	**0.2999**
25	0.3586	0.2451	0.2912
30	0.3400	0.2413	0.2823
35	0.3395	0.2378	0.2797

**Table 6:** Performance comparison of the no-candidate herbal set, the single-candidate herbal set, and the double-candidate herbal set.

Models	Precision	Recall	F1-score
noCHL-noHCP-GSCCAM	0.3155	0.2005	0.2452
Only-CHL-GSCCAM	0.3292	0.2201	0.2638
Only-HCP-GSCCAM	0.3536	0.2439	0.2887
GSCCAM	**0.3739**	**0.2504**	**0.2999**

**Table 7:** Comparison of predicted cases among labeled prescriptions, baseline models and our model (GSCCAM), and their human qualitative evaluation scores. Herbs whose predicted prescriptions are consistent with the labeled prescriptions are marked in bold font, and herbs that appear in the candidates are underlined.

	Case 1		Case 2	
Symptoms	Sixty-four kinds of wind enter skin, crawling like insects, stiff waist and spine, disharmony of internal organs, inability to stretch hands and feet, urticaria turns into sores after being scratched, itches on the body when the wind blows, finally, the face swells, the hands cannot be raised over the head, the mouth cannot talk.	Total	The Yang Qi in the liver is weak, the yin and cold are condensed, the ribs under the armpit are painful and full of Qi, and the eyes are blurred.	Total
Label prescription	Photinia serratifolia, Dried Zingiber, Scutellaria baicalensis georgi, Asarum, dried Rehmannia glutinosa, Ligusticum chuanxiong Hort, Panax ginseng, ephedra, peeled cinnamomum cassia, Angelica sinensis, Glycyrrhiza, Zanthoxylum ailanthoides.	8.5	Areca catechu, old zingiber, Aconitum carmichaeli, Poria cocos, Evodia rutaecarpa, Pericarpium Citri reticulata, peeled cinnamomum cassia, Platycodon grandiflorus, Atractylodes macrocephala.	8.8
KDHR [18]	**Photinia serratifolia**, Saposhnikovia divaricata, Anemarrhena asphodeloides Bunge, Akebia quinata, Cynanchum otophyllum, Poria cocos, Cimicifuga foetida, dendrobium, Bupleurum, Gardenia jasminoides, Rehmannia glutinosa, Ziziphus jujuba, Atractylodes macrocephala, Houpoea officinalis, Mentha, Pueraria lobata, Citrus reticulata, Tribulus terrestris, Panax notoginseng, Arctium lappa, Fallopia multiflora.	3.9	**Areca catechu**, **Aconitum carmichaeli**, Cinnamomum cassia, Dimocarpus longan, roasted Zingiber, curcuma, Bupleurum, dried Zingiber, Dendrobium, Panax ginseng, Citrus medica, Perilla frutescens, chrysanthemum, Mentha, Houpoea officinalis.	4.3
PTM [21]	**Photinia serratifolia,** Zingiber, Saposhnikovia divaricata, Perilla frutescens, Phellodendron, corydalis yanhusuo, Corydalis decumbens, Coptis chinensis, curcuma, roasted Zingiber.	5.1	**Areca catechu**, Zingiber, Cinnamomi Ramulus, dried Zingiber, Eucommia ulmoides, Cinnamomum cassia, Lycium barbarum.	5.5
Coverage-Soft-Model [44]	**Photinia serratifolia**, Phellodendron, folium pini, aconitum kusnezoffii, Arctium lappa, Codonopsis pilosula, Mylabris, Aconitum carmichaeli, Bambusa basihirsuta McClure.	3.5	**Areca catechu**, Zingiber, Syzygium aromaticum, Cinnamomum cassia, Curcuma, Ephedra, Asarum.	4.0
Herb-Know [24]	**Photinia serratifolia**, **Scutellaria baicalensis georgi**, Anemarrhena asphodeloides Bunge, Asarum, Atractylodes macrocephala, roasted Zingiber, Achyranthes bidentata, Corydalis decumbens, Saposhnikovia divaricata, Coptis chinensis, Tribulus terrestris, Alumen, Pinellia ternate, Nepeta cataria, Pericarpium Citrus reticulata.	5.7	**Areca catechu**, Zingiber, **Aconitum carmichaeli**, **Poria cocos**, Cynanchum otophyllum, Astragalus membranaceus, Eucommia ulmoides, Citrus medica, Lycium barbarum, Cassia tora, Dendrobium.	5.6
TPGen [23]	**Photinia serratifolia**, Zingiber, **Scutellaria baicalensis georgi**, Pinellia ternate, Ziziphus jujuba, Pericarpium Citrus reticulata, **Angelica sinensis**, Corydalis yanhusuo, Dendrobium, Panax notoginseng.	5.4	**Areca catechu**, Lycium barbarum, dried zingiber, **Aconitum carmichaeli**, **Pericarpium Citri reticulata**, Ziziphus jujuba, Dioscoreae, aconitum.	5.8
GSCCAM	**Photinia serratifolia**, Cinnamomi Ramulus, **Angelica sinensis**, **Glycyrrhiza**, Zingiber, Nepeta cataria, **Scutellaria baicalensis georgi**, **Asarum**, **Panax ginseng**, **dried Rehmannia glutinosa**, **Zanthoxylum ailanthoides**, Saposhnikovia divaricata, Angelica dahurica.	7.6	**Areca catechu**, **Platycodon grandiflorus**, Zingiber, **Aconitum carmichaeli**, **Poria cocos**, Cinnamomi Ramulus, **Pericarpium Citri reticulata**, Astragalus membranaceus, **Evodia rutaecarpa**.	7.8

## Data Availability

The datasets used to support the findings of this study are available from the corresponding author upon request.

## References

[1] Fu L., Wang Y. R., Zhao Y. P., Huang L. Q. (2022). The concept of traditional Chinese medicine: history, theory and empirical research. *Zhonghua yi shi za zhi*.

[2] Lin S., Yan Y., Chenxi H. (2022). Influence of degree of Chinese cultural identity and performance expectation on consumption attitude for health care of traditional Chinese medicine. *Computational and Mathematical Methods in Medicine*.

[3] Zhang S.-Q., Li J.-C. (2021). An introduction to traditional Chinese medicine, including acupuncture. *The Anatomical Record*.

[4] Li B., Despotova-Toleva L. (2021). Practice and thinking of modern clinical research of traditional Chinese medicine. *Annals of palliative medicine*.

[5] Zhang W., Zhao Y., Liu H., Jing C. (2022). Efficacy of traditional Chinese medicine combined with chemotherapy in the treatment of gastric cancer: a meta-analysis. *Computational and Mathematical Methods in Medicine*.

[6] Zhi W., Liu Y., Wang X., Zhang H. (2023). Recent advances of traditional Chinese medicine for the prevention and treatment of atherosclerosis. *Journal of Ethnopharmacology*.

[7] Chennan W., Chen F., Huang S. (2023). Progress on the role of traditional Chinese medicine in therapeutic angiogenesis of heart failure. *Journal of Ethnopharmacology*.

[8] Wei Z., Chen J., Zuo F. (2023). Traditional Chinese medicine has great potential as candidate drugs for lung cancer: a review. *Journal of Ethnopharmacology*.

[9] Ren J.-l., Zhang A.-H., Wang X.-J. (2020). Traditional Chinese medicine for covid-19 treatment. *Pharmacological Research*.

[10] Zhao Z., Li Y., Zhou L. (2021). Prevention and treatment of covid-19 using traditional Chinese medicine: a review. *Phytomedicine*.

[11] Ren X., Shao X.-X., Li X.-X. (2020). Identifying potential treatments of covid-19 from traditional Chinese medicine (tcm) by using a data-driven approach. *Journal of Ethnopharmacology*.

[12] Wang W.-Y., Zhou H., Wang Y.-F., Sang B.-S., Liu L. (2021). Current policies and measures on the development of traditional Chinese medicine in China. *Pharmacological Research*.

[13] Shi X., Zhu D., Nicholas S., Hong B., Man X., He P. (2020). Is traditional Chinese medicine “mainstream” in China? Trends in traditional Chinese medicine health resources and their utilization in traditional Chinese medicine hospitals from 2004 to 2016. *Evidence-based Complementary and Alternative Medicine*.

[14] Zhang Y., Zhao J., Qiang Y., Yang X., Wei W., Jia L. (2022). Improved heterogeneous data fusion and multi-scale feature selection method for lung cancer subtype classification. *Concurrency and Computation: Practice and Experience*.

[15] Qin Y., Ma Z. (2020). A traditional Chinese medicine prescription recommendation method based on mutual information clustering. *Journal of Physics: Conference Series*.

[16] Shi Q. Y., Tan L. Z., Seng L. L., Wang H. J. (2021). Intelligent prescription-generating models of traditional Chinese medicine based on deep learning. *Journal of Traditional Chinese Medicine*.

[17] Jin Y., Zhang W., He X., Wang X., Wang X. Syndrome-aware herb recommendation with multi-graph convolution network.

[18] Yang Y., Rao Y., Minghao Y., Kang Y. (2022). Multi-layer information fusion based on graph convolutional network for knowledge-driven herb recommendation. *Neural Networks*.

[19] Zhao W., Weikai L., Li Z. (2022). Tcm herbal prescription recommendation model based on multi-graph convolutional network. *Journal of Ethnopharmacology*.

[20] Wang X., Zhang Y., Wang X., Chen J. (2019). A knowledge graph enhanced topic modeling approach for herb recommendation. *International Conference on Database Systems for Advanced Applications*.

[21] Yao L., Zhang Y., Wei B., Zhang W., Jin Z. (2018). A topic modeling approach for traditional Chinese medicine prescriptions. *IEEE Transactions on Knowledge and Data Engineering*.

[22] Sutskever I., Vinyals O., Le Q. V. (2014). *Sequence to sequence learning with neural networks*. *Advances in neural information processing systems*.

[23] Ruan C., Luo H., Yingpei W., Yang Y. (2021). *Tpgen: Prescription generation using knowledge-guided translator*.

[24] Li C., Liu D., Yang K., Huang X., Lv J. Herb-know: knowledge enhanced prescription generation for traditional Chinese medicine.

[25] Wang Z., Poon J., Poon S. Tcm translator: a sequence generation approach for prescribing herbal medicines.

[26] Liu Z., Zheng Z., Guo X. (2019). Attentiveherb: a novel method for traditional medicine prescription generation. *IEEE Access*.

[27] Yang W., Dong Y., Qianqian D. (2021). Integrate domain knowledge in training multi-task cascade deep learning model for benign--malignant thyroid nodule classification on ultrasound images. *Engineering Applications of Artificial Intelligence*.

[28] Shi G., Wang J., Qiang Y. (2020). Knowledge-guided synthetic medical image adversarial augmentation for ultrasonography thyroid nodule classification. *Computer Methods and Programs in Biomedicine*.

[29] Ma Y., Wang J., Song K., Qiang Y., Jiao X., Zhao J. (2021). Spatial-frequency dual-branch attention model for determining *KRAS* mutation status in colorectal cancer with T2-weighted MRI. *Computer Methods and Programs in Biomedicine*.

[30] Wang J. W., Cui Y. F., Shi G. H. (2020). Multi-branch cross attention model for prediction of KRAS mutation in rectal cancer with t2-weighted MRI. *Applied Intelligence*.

[31] Jiasen L., Batra D., Parikh D., Lee S. (2019). Vilbert: pretraining task-agnostic visiolinguistic representations for vision-and-language tasks. *Advances in Neural Information Processing Systems*.

[32] Wang L., Li Y., Huang J., Lazebnik S. (2019). Learning two-branch neural networks for image-text matching tasks. *IEEE Transactions on Pattern Analysis and Machine Intelligence*.

[33] Chen W., Jiang H., Wu Q., Karlsson B. F., Guan Y. (2021). Advpicker: effectively leveraging unlabeled data via adversarial discriminator for cross-lingual ner.

[34] Chen J., Dai X., Yuan Q., Lu C., Huang H. Towards interpretable clinical diagnosis with Bayesian network ensembles stacked on entity-aware cnns.

[35] Deng Z., Ma F., Lan R., Huang W., Luo X. (2021). A two-stage chinese text summarization algorithm using keyword information and adversarial learning. *Neurocomputing*.

[36] Bahdanau D., Cho K., Bengio Y. (2014). Neural machine translation by jointly learning to align and translate.

[37] Liu Z., Luo C., Dianzheng F. (2022). A novel transfer learning model for traditional herbal medicine prescription generation from unstructured resources and knowledge. *Artificial Intelligence in Medicine*.

[38] Yao N., Zhu J., Gao R. (2013). *Traditional Chinese medicine symptoms differential diagnosis*.

[39] Cho K., Van Merriënboer B., Bahdanau D., Bengio Y. (2014). On the properties of neural machine translation: Encoder-decoder approaches.

[40] Liu J.-X., Ren J.-X., Lin C.-R. (2016). Research and development on efficacy of Chinese herbal compound. Zhongguo Zhong yao za zhi= Zhongguo Zhongyao Zazhi= China journal of Chinese Materia. *Médica*.

[41] Qiao Y., Zhang Y., Peng S. (2022). Property theory of Chinese materia medica: clinical pharmacodynamics of traditional Chinese medicine. *Journal of Traditional Chinese Medical Sciences*.

[42] Radford A., Wu J., Child R., Luan D., Amodei D., Sutskever I. (2019). Language models are unsupervised multitask learners. *Open AI blog*.

[43] Li F. (2021). Identifying the monarch medicine in formulas. *Journal of Nanjing University of Traditional Chinese Medicine*.

[44] Li W., Yang Z. (2019). Exploration on generating traditional Chinese medicine prescriptions from symptoms with an end-to-end approach. *CCF International Conference on Natural Language Processing and Chinese Computing*.

[45] Verboven S., Chaudhary M. H., Berrevoets J., Verbeke W. (2020). Hydalearn: Highly dynamic task weighting for multi-task learning with auxiliary tasks.

